# Co-cultivation strategies for natural product discovery from actinomycetes: unlocking silent secondary metabolism with mycolic acid-containing bacteria

**DOI:** 10.1007/s11274-025-04406-7

**Published:** 2025-06-25

**Authors:** Shumpei Asamizu

**Affiliations:** Engineering Biology Research Center, Kobe University, 1-1 Rokkodai, Nada, Kobe, 657-8501 Japan

**Keywords:** Co-culture, Combined-culture, Natural product discovery, Bacterial interaction, Actinomycetes, Secondary metabolism

## Abstract

Bacteria form consortia as integral components of diverse ecosystems, where they interact with various organisms. Within these communities, bacterial–bacterial communication plays a pivotal role by driving numerous specific interactions. A key aspect of this chemical communication is the production of secondary metabolites. Recent research demonstrates that interspecies interactions between microorganisms can serve as physiological triggers, activating silent biosynthetic gene clusters and leading to the synthesis of novel secondary metabolites by the interacting species. This review focuses on mixed cultivation strategies involving actinobacteria, with an emphasis on utilizing mycolic acid-containing bacteria such as *Tsukamurella pulmonis* as inducer organisms. It comprehensively examines recent advances striving to understand these bacterial interactions, specifically involving the ability of actinomycetes to recognize and respond to mycolic acid-containing bacteria to activate secondary metabolism. Furthermore, the genetic basis of secondary metabolism activation was explored and newly discovered secondary metabolites induced by actinobacteria–mycolic acid-containing bacteria co-culture were highlighted. Finally, the integration of combined-culture strategies with genetic engineering methods and the ecological relevance of actinobacteria–mycolic acid-containing bacteria interactions were discussed. These bacterial interactions provide an excellent model system for understanding the molecular mechanisms regulating secondary metabolism and could open new avenues for drug discovery.

## Introduction

Actinomycetes, particularly those in the genus *Streptomyces*, are filamentous soil bacteria renowned for producing a diverse array of natural products through secondary metabolism (Hutchings et al. 2019; Parra et al. 2023). It is estimated that over 60% of all discovered practically used bioactive natural products originate from these bacteria (Berdy 2005; Hutchings et al. 2019). These compounds have served as lead molecules for drug discovery in pharmaceuticals and agrochemicals, significantly contributing to human advancement (Newman and Cragg 2020). However, traditional discovery methods often lead to the rediscovery of known compounds, making it challenging to identify novel natural products with unique chemical structures and unprecedented targets (Newman and Cragg 2020). Meanwhile, the rising threat of antibiotic-resistant bacteria and the increasing spread of infectious diseases underscores the urgent need for the development of new antibiotics (O'Neill et al. 2016). Consequently, the significance of actinomycete-derived natural products continues to grow.

Since the 2000 s, advances in next-generation sequencing technology have revealed the genomes of numerous microorganisms, including model actinomycetes such as *Streptomyces coelicolor* A3(2) (Bentley et al. 2002). This was followed by the sequencing of industrially important antibiotic producers, including *Streptomyces avermitilis* (macrocyclic type-I polyketide anthelmintic drug avermectin) (Ikeda et al. 2003), *Streptomyces griseus* (aminoglycoside antibiotic streptomycin) (Ohnishi et al. 2008), and *Saccharopolyspora erythraea* (macrocyclic type-I polyketide antibiotic erythromycin) (Oliynyk et al. 2007). Currently, approximately 200 complete genome sequences of *Streptomyces* species are available in the Kyoto Encyclopedia of Genes and Genomes (KEGG) genome database (https://www.genome.jp/kegg/genome/). Genome analysis has predicted over 30 biosynthetic gene clusters (BGCs) involved in secondary metabolism in each actinomycete genome. This was surprising at the time, as the number of specific metabolites obtained from a single strain was generally fewer than five (Nett et al. 2009). More recently, large-scale genome sequence analyses strongly suggest that bacteria such as actinomycetes harbor over 10,000 potential BGCs for secondary metabolites (SMs). These clusters are predicted to encode numerous undiscovered natural products with novel chemical scaffolds (Navarro-Munoz et al. 2020; Gavriilidou et al. 2022). However, their detection and characterization is challenging because most of these potential SM BGCs remain silent (non-expressive) or produce minute quantities of metabolites. Despite the vast genetic potential revealed by genome sequencing, an effective strategy for systematically activating these cryptic BGCs remains elusive. Overcoming this challenge is crucial for unlocking novel natural products. Recently, new approaches have emerged, including genetic strategies using CRISPR/Cas-based activation (Tong et al. 2019) and synthetic biology techniques (Rice et al. 2025; Seshadri et al. 2025).

Concurrently, it is increasingly recognized that bacteria form consortia that are essential components of diverse ecosystems, where they interact with various organisms (Wang et al. 2024). Within these communities, inter-bacterial communication plays a crucial role, facilitating numerous specific interactions. SMs are key players in this chemical communication. This review explores the activation of secondary metabolism in mixed cultivation, with a particular focus on actinobacteria and mycolic acid-containing bacteria (MACB) as inducer organisms. This review comprehensively describes the specific interaction between filamentous soil-dwelling *Streptomyces* species and *Tsukamurella pulmonis* TP-B0596 (a MACB) at the molecular level, along with the characteristics of the induced secondary metabolism.

### MACB as efficient co-culture partners for inducing secondary metabolism in actinobacteria

Many bioactive natural products produced as SMs play a crucial role in chemical communication within bacterial communities (Traxler and Kolter 2015; van Bergeijk et al. 2020; Wang et al. 2024). The potential of co-culturing microorganisms has been proposed and tested as a strategy for screening the production of new SMs (Bertrand et al. 2014; Hoshino et al. 2019). A pioneering study in this field was conducted by the Fenical group, which explored interactions between marine fungi and bacteria to identify specific metabolites produced in co-culture (Cueto et al. 2001). Since that time, the rapid accumulation of genome sequencing data in the 2000 s has revealed numerous cryptic BGCs encoding novel molecular skeletons within microbial genomes (Gavriilidou et al. 2022). This has drawn increasing attention to strategies for activating these silent or poorly expressed genes for secondary metabolism to facilitate natural product discovery. Today, bacterial co-culture is widely recognized as a key approach for uncovering novel metabolites (Bertrand et al. 2014; Hoshino et al. 2019).

**Fig. 1:**
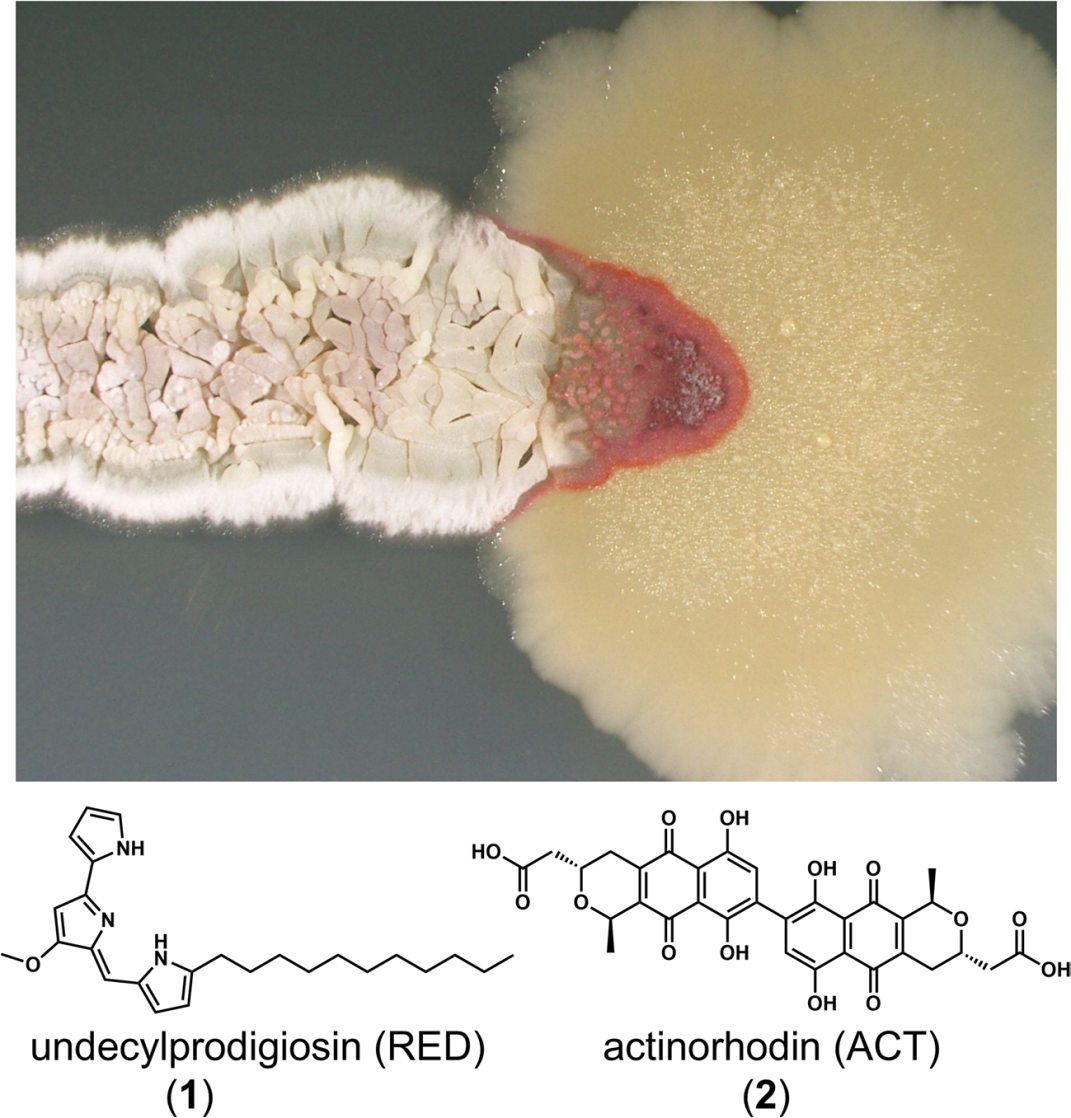
Dual-culture of *Streptomyces lividans* TK23 and *Tsukamullera pulmonis* TP-B0596 on an agar plate. Highly induced production of RED is observed at the edge of the colony

In view of this background, Onaka et al. (2011) reported a bacterium capable of inducing SM production in actinomycetes. *Streptomyces lividans* TK23 was used as the indicator strain, as it conditionally produces the red pigment undecylprodigiosin (RED) (**1**) (Williamson et al. 2006), allowing for a visually straightforward evaluation of RED production activation (Fig. 1). Newly isolated soil bacteria were co-cultured with *S. lividans* to screen for strains capable of inducing RED production, with *Tsukamurella pulmonis* TP-B0596 found to effectively induce RED production (Onaka et al. 2011). The *Tsukamurella* genus belongs to the Actinomycetes class and the Mycobacteriales order, and contains mycolic acids (C30–C90 fatty acids) in its cell wall (Marrakchi et al. 2014). Using *T. pulmonis* TP-B0596 as an inducer strain, 42 new natural products were isolated from 16 actinobacterial strains, including rare genera such as *Actinosynnema*, *Micromonospora*, *Umezawaea*, *Catenuloplanes*, *Amycolatopsis*, and *Saccharothrix* (Table 1). This makes *T. pulmonis* TP-B0596 one of the most successful inducer bacteria for SM production in antinomycetes.

**Table 1:** Natural products isolated from actinomycetes vs. MACB co-culture

producing strain	inducer strain	induced natural products	bioactivity	reference(s)
*Streptomyce lividans* TK23	Tp	RED (**1**) and ACT (**2**)		(Onaka et al. 2011)
*Streptomyces coelicolor* JCM4020	Tp	RED (**1**)		(Yanagisawa et al. 2022)
*Streptomyces coelicolor* A3(2)	Tp	RED (**1**)		(Lei et al. 2024)
*Micromonospora* sp. WMMB235	*Rhodococcus* sp. WMMA185	keyicin (**3**)	antibacterial	(Adnani et al. 2017)
*Streptomyces nigrescens* HEK616	Tp	5-alkyl-1,2,3,4-tetrahydroquinolines (5aTHQs) (**4**–**11**)	antifungal	(Sugiyama et al. 2015)
*Streptomyces nigrescens* HEK616	Tp	streptoaminals (**12**–**14**)	antibacterial	(Sugiyama et al. 2016)
*Streptomyces* sp. NZ-6	Tp	niizalactams A, B and C (**15**–**17**)		(Hoshino et al. 2015a)
*Actinosynnema mirum* NBRC14064	Tp	mirilactams C, D and E (**18**–**20**)		(Hoshino et al. 2018b)
*Micromonospora wenchangensis* HEK797	Tp	dracolactams A and B (**21**, **22**)		(Hoshino et al. 2017)
*Umezawaea* sp. RD066910	Tp	umezawamides A and B (**23**, **24**)	cytotoxic	(Hoshino et al. 2018c)
*Nocardiopsis* sp. FU40 ΔApoS	Rhodococcus wratislaviensis	ciromicins A and B (**25**, **26**)		(Derewacz et al. 2015)
*Catenuloplanes* sp. RD067331	Tp	catenulobactins A and B (**27**, **28**)	siderophore	(Hoshino et al. 2018a)
*Streptomyces davawensis* JCM4913	Tp	desferrioxamine I1a, I1b and I2a (**29**–**31**)	siderophore	(Hagihara et al. 2018)
*Streptomyces hygroscopicus* HOK021	Tp	harundomycin A (**32**)	antibacterial/siderophore	(Asamizu et al. 2022)
*Streptomyces* sp. KUSC_F05	Tp	longicatenamides A, B, C, and D (**35**–**38**)	antibacterial	(Jiang et al. 2021)
*Amycolatopsis* sp. 26–4	Tp	amycolapeptins A and B (**33**, **34**)		(Pan et al. 2021)
*Amycolatopsis* sp. 26–4	Tp	amoxetamide A (**39**)	anoikis inducer	(Pan et al. 2024)
*Streptomyces endus* S-522	Tp	alchivemycin A (**40**)	antibacterial	(Igarashi et al. 2010)
*Streptomyces* sp. CJ-5	Tp	chojalactones A, B and C (**41**–**43**)	cytotoxic	(Hoshino et al. 2015b)
*Streptomyces tendae* KMC006	Gordonia sp. KMC005	gordonic acid (**45**)		(Park et al. 2017)
*Streptomyces cinnamoneus* NBRC13823	Tp	arcyriaflavin E (**46**)	cytotoxic	(Hoshino et al. 2015c)
*Saccharothrix* sp. A1506	Tp	saccharothriolide C-2 (**48**)		(Jiang et al. 2019)

Tp: *Tsulamurella pulmonis* TP-B0596

### What does the SM-producing actinomycete recognize in the co-culture?

For many years, modifying nutrient components or compositions was a widely used strategy, since it effectively enhanced or induced SMs production in actinobacteria. The availability and balance of carbon and nitrogen sources, which are often limited in the soil environment, significantly influence the production of SMs. Additionally, stress responses triggered by factors such as antibiotics, pH shifts, oxidative stress, or specific cell components like N-acetylglucosamine have also been shown to stimulate the production of SMs (van der Heul et al. 2018). Therefore, the following aspects should be considered to play a role in inducing SM production in bacterial co-culture: nutrient exchange and competition, stress responses, and general bacterial communication (including signaling molecules and quorum sensing). Within these circumstances, investigating the specific factors (if any) that induce SM production will be crucial for developing a simpler and more stable method to trigger secondary metabolism in actinobacteria.

Along with the discovery of *T. pulmonis* as a strong inducer of SM production in actinobacteria, other genera within Mycobacteriales were tested for their ability to induce RED production in *S. lividans*. A group of bacteria with mycolic acids in their cell walls, referred to as MACB, exhibits strong inducing activity (Onaka et al. 2011). Although the involvement of mycolic acids as chemical inducers was speculated due to their specificity in MACB, purified mycolic acid did not induce RED production (Onaka et al. 2011). Interestingly, RED production was not induced when *S. lividans* and *T. pulmonis* cultures were separated by a dialysis membrane, suggesting that direct cell-to-cell contact is essential for secondary metabolism induction (Onaka et al. 2011). Scanning electron microscopy (SEM) revealed significant structural interactions between both cells (Asamizu et al. 2015). To test whether *S. lividans* recognizes continuous exposure to the complex cell wall architecture associated with mycolic acids, dead MACB cells were prepared using formaldehyde fixation or γ-ray treatment, which preserves cell architecture. However, these dead cells failed to induce RED production (Asamizu et al. 2015). Consequently, secondary metabolism activation in actinomycetes by MACB is suggested to involve a complex interplay of factors triggered by multiple stimuli from living cells. The 4.67 Mb genome of *T. pulmonis* TP-B0596 has been sequenced (GenBank No.: AP025457), providing valuable data for future research on the molecular mechanisms by which it stimulates SM production (Asamizu et al. 2022).

**Fig. 2:**
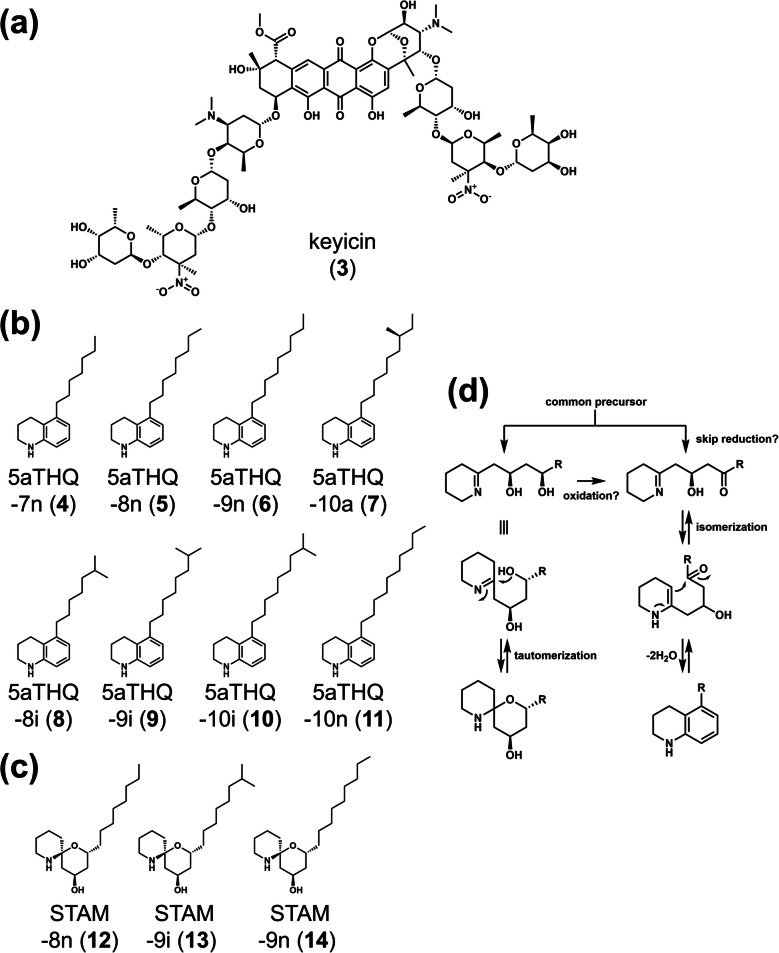
Chemical structures of SMs induced in actinobacteria during co-culture with MACB. Chemical structure of keyicin from a co-culture of marine derived *Micromonospora* sp. WMMB235 and *Rhodococcus* sp. WMMA185 (**a**). Chemical structure of 5aTHQs (**b**) and streptoaminals (STAMs) (**c**) from a combined-culture of *S. nigrescens* HEK616 and *T. pulmonis* TP-B0596. The numbers in the compound names refer to the carbon length of the acyl chains. The prefixes “n,” “i,” and “a” denote normal-type, iso-type, and anteiso-type branching, respectively. Putative biosynthetic pathway for 5aTHQs and streptoaminals from the common precursor (**d**)

Bugni and his colleagues isolated keyicin (**3**) (Fig. 2a), a highly glycosylated anthracycline antibiotic from a co-culture of *Micromonospora* sp. WMMB235 and *Rhodococcus* sp. WMMA185 (MACB) (Adnani et al. 2017). Interestingly, keyicin exhibited selective growth-inhibitory activity against Gram-positive bacteria, including *Rhodococcus* species, suggesting a competitive interaction between the producing strain and its microbial neighbors. Unlike the interaction between *S. lividans* and *T. pulmonis* for the production of RED, keyicin production was observed even when the two strains were separated by a dialysis membrane, which prevented direct cell-to-cell contact but allowed the diffusion of small molecules (Adnani et al. 2017). The genome of strain WMMB235 was sequenced, revealing the presence of a conserved homolog of acyl-homoserine lactone (AHL) receptor protein (Acharya et al. 2019). The quorum sensing mechanism in the inducer MACB (including *Rhodococcus* sp. WMMA185) remains unknown, although the presence of this unusual AHL receptor protein in the *Micromonospora* sp. genome led to the hypothesis that AHL-mediated chemical communication between the two bacteria might activate keyicin production. However, no significant evidence supports the involvement of AHL in this interaction, and the mechanism of keyicin activation remains unclear (Acharya et al. 2019). This result shows that the factors inducing secondary metabolism in the co-culture system appear to involve independent mechanisms for each SM.

### Genes involved in the activation of SMs production induced by actinobacteria vs. MACB co-culture

Investigating the molecular mechanisms by which actinobacteria respond to stimuli via MACB is important, as it may provide a genetic tool to rationally activate silent SMs production. Actinobacteria found in soil environments generally have genomes larger than 8 Mb, containing around 8,000 genes or more (Nett et al. 2009). The genome typically contains more than 30 biosynthetic genes for SMs (Nett et al. 2009; Gavriilidou et al. 2022), as well as putative regulatory proteins (Pei et al. 2024), transporters (Severi and Thomas 2019), and hypothetical proteins (Lei et al. 2023, 2024) that may be involved in the response regulatory systems required to adapt to the complex soil ecosystem, which contains many organisms. Additionally, investigating molecular mechanisms may reveal genes involved in previously unknown regulatory systems that interact with specific factors.

To identify genes involved in co-culture-induced activation of RED production in model actinomycetes, a forward genetic study was conducted using heavy ion (^12^C^5+^) beam-induced mutagenesis in *S. coelicolor* JCM4020 (Yanagisawa et al. 2022). Screening for mutants defective in RED production identified 86 mutants capable of growing on minimal medium from approximately 152,000 irradiated spores (Yanagisawa et al. 2022). Among these mutants, inactivation of *sco1842*, designated as *ccr1* (combined-culture-related regulatory protein no. 1), resulted in a *S. coelicolor* A3(2) strain with reduced production of RED and several other SMs (Lei et al. 2024). The gene product was hypothesized to be a nucleotide-associated protein (NAP), as it exhibited weak homology to a helix–turn–helix motif and a helicase C-terminal domain (Lei et al. 2024). The *ccr1* gene was transcriptionally upregulated in the combined culture, and its homolog is highly conserved among *Streptomyces* species. In addition to strain A3(2), the production of streptoaminals (STAM) (**12–14**) and 5-alkyl-1,2,3,4-tetrahydroquinolines (5aTHQs) (**4–11**) in *Streptomyces nigrescens* HEK616 (detail provided later) was diminished by the disruption of the *ccr1* homolog (Lei et al. 2024). These results indicate that *ccr1* is one of the key genes involved in secondary metabolism activation in the co-culture system. Other than *ccr1*, a forward genetic study revealed that the over-expression of multidrug efflux pumps (encoded by *sco1718-20*, *sco4358-4360*, and *sco5384-5382*, with each regulated by a dedicated TetR-type repressor) was associated with reduced RED production (Lei et al. 2023).

### Secondary metabolism induced by actinobacteria vs. MACB co-culture

#### Highly reductive type II-like polyketides

Eight congeners of 5-alkyl-1,2,3,4-tetrahydroquinolines (5aTHQs), each with different alkyl chains, were isolated from a combined-culture of *S. nigrescens* HEK616 and *T. pulmonis* (Sugiyama et al. 2015) (Fig. 2b). Hereafter, co-culture involving actinomycetes and MACB for the activation of SM production will be referred to as"combined-culture"(Hoshino et al. 2019). The 5aTHQs exhibited antifungal activity against *Schizosaccharomyces pombe*, and it is predicted that they interact with ergosterol in the fungal cell membrane. The congeners with different alkyl chains exhibited unique biological properties. A congener with a long alkyl chain (e.g., 5aTHQ-10n) did not exhibit antifungal activity on its own. However, when mixed in equal parts (0.5/0.5) with a congener possessing a short alkyl chain (e.g., 5aTHQ-7n), the mixture exhibited antifungal activity comparable to that of 5aTHQ-7n alone (Sugiyama et al. 2019). These results suggest that the inherent flexibility in the biosynthetic pathway is functionally advantageous, as it leads to structural variations, with the resulting metabolites exhibiting synergistic activity. This"sloppiness"can be considered advantageous for interactions in complex ecosystems containing a variety of microorganisms.

Three congeners of streptoaminals, each containing a [5,5]-spirohemiaminal ring with varying alkyl chains, were also isolated from the combined culture of *S. nigrescens* HEK616 and *T. pulmonis* (Sugiyama et al. 2016) (Fig. 2c). Investigation of the biosynthetic genes responsible for the production of 5aTHQs and streptoaminals identified a gene cluster comprising nine genes in the genome of *S. nigrescens* HEK616, including two sets of ketosynthase/chain length factor (KS/CLF) and an aminotransferase. Heterologous expression of this BGC forming an operon (*stm* BGC) in *S. lividans* TK23 under a strong constitutive promoter produced streptoaminals. Interestingly, the production of 5aTHQs was also observed when heterologous expression of the *stm* BGC was united with the combined-culture. It is predicted that either skipping the dehydrolase reaction of the common precursor or re-oxidation of streptoaminal may generate the precursor for 5aTHQ production (Fig. 2d). Since 5aTHQ production is highly specific to combined-culture, the predicted biosynthetic route may be specifically induced in the co-culture system or result from conversion by the partner MACB.

#### Polyene macrolactams

**Fig. 3:**
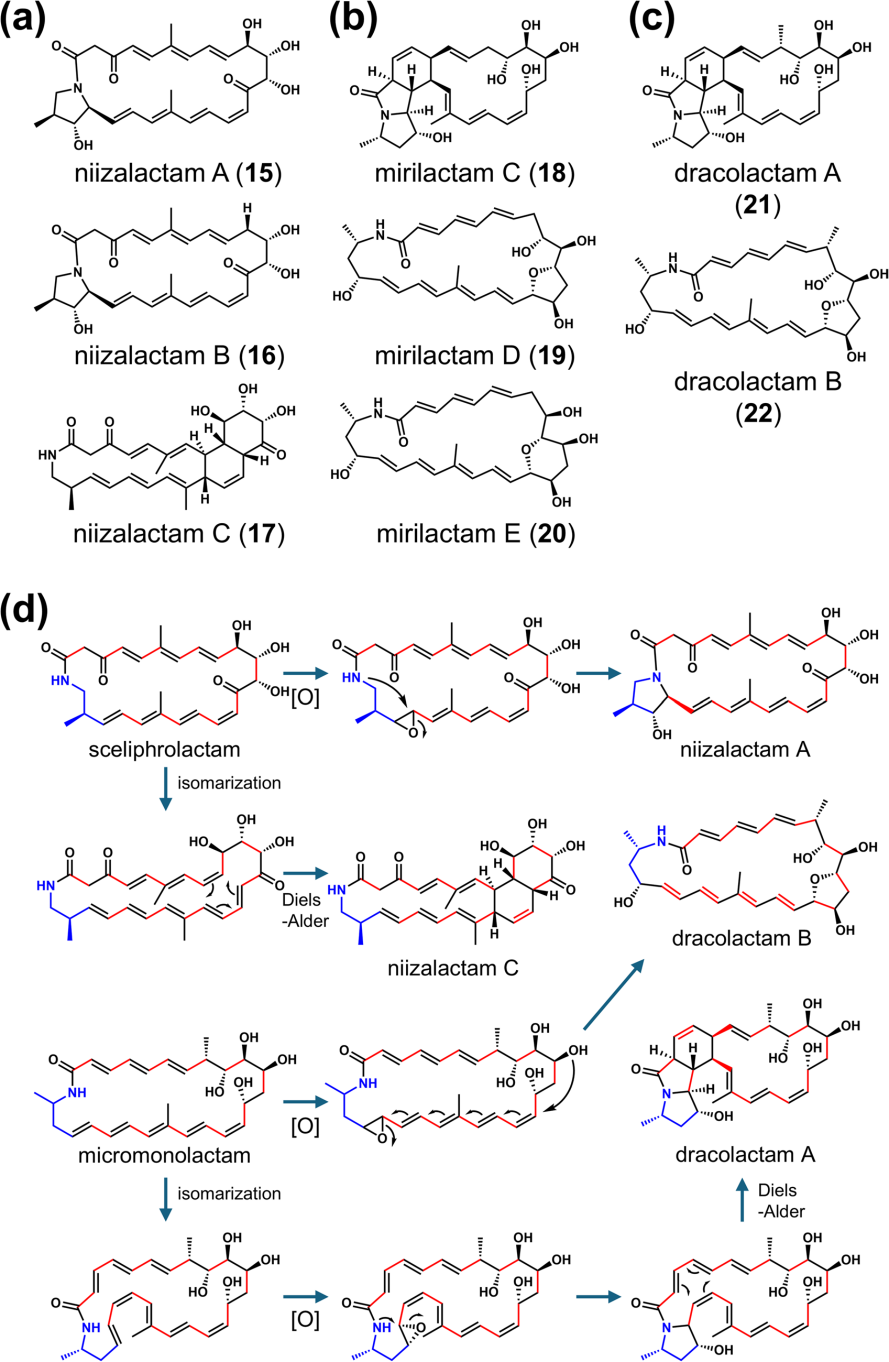
Chemical structures and derivatization pathway of polyene macrolactams induced in actinobacteria during co-culture with MACB. Chemical structures of polyene macrolactams: niizalactams A–C (**a**), mirilactams C–E (**b**), and dracolactams A–B (**c**) from a combined-culture with *T. pulmonis* TP-B0596. Putative biosynthetic pathway for macrolactam derivatization from a common precursor, involving *E*/*Z* isomerization, epoxidation, and Diels–Alder cycloaddition reactions induced in the combined-culture (**d**)

**Fig. 4:**
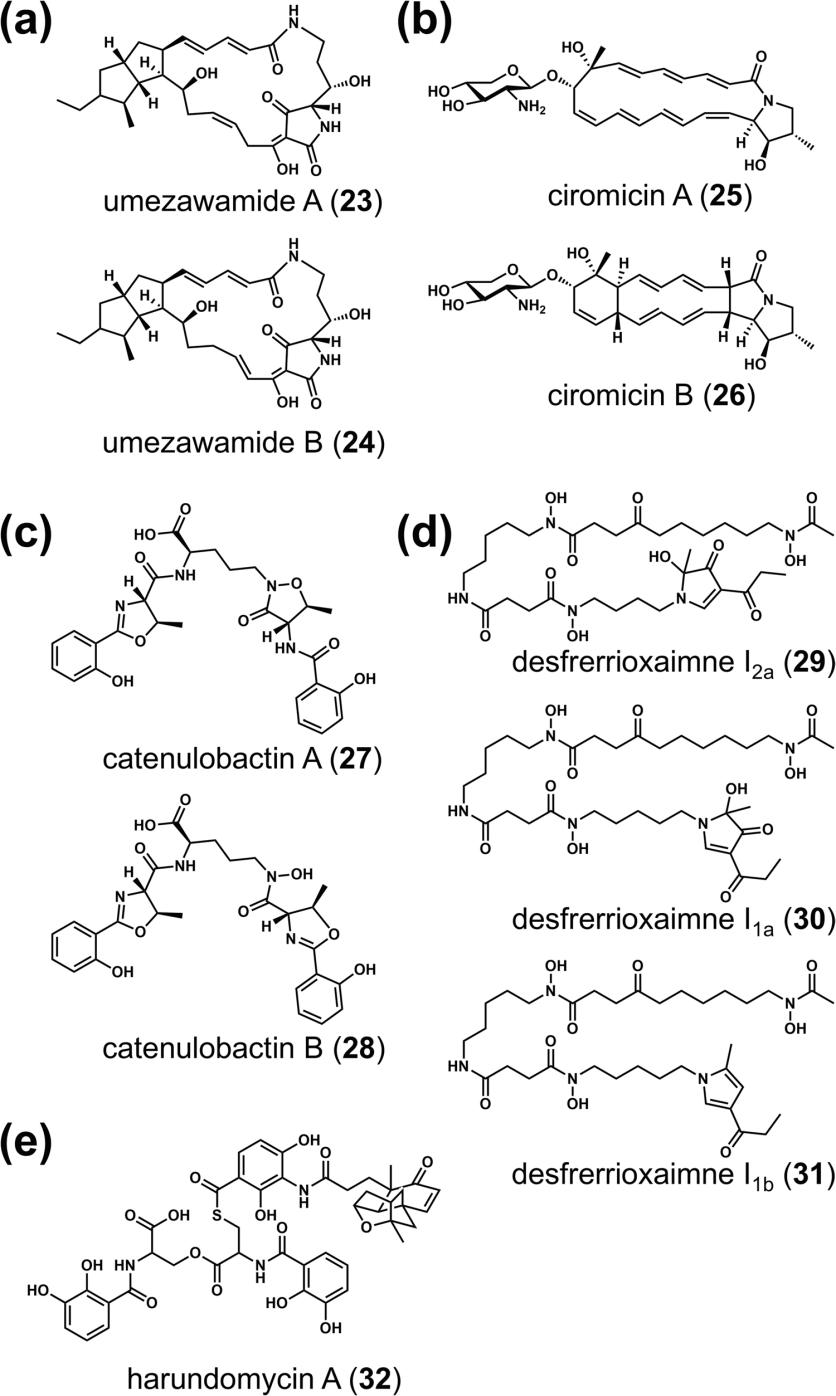
Chemical structures of polyene macrolactams and siderophores induced in actinobacteria during co-culture with MACB. Chemical structures of polyene macrolactams: umezawamides A–B from a combined-culture with *T. pulmonis* TP-B0596 (**a**). Chemical structures of ciromicins A–B from co-culture of *Nocardiopsis* sp. FU40 ΔApoS and *Rhodococcus wratislaviensis* (**b**). Chemical structures of siderophores induced in combined-culture: catenulobactin A–B (**c**), desfrerrioxaimnes I_2a_, I_1a_, and I_1b_ (**d**), and siderophore-antibiotic conjugate harundomycin A (**e**)

Polyene macrolactams with different cyclization patterns were isolated from combined-cultures with *T. pulmonis*. Niizalactams A–C (**15–17**) were discovered in *Streptomyces* sp. NZ-6 (Hoshino et al. 2015a) (Fig. 3a), mirilactams C–E (**18–20**) in *Actinosynnema mirum* NBRC 14064 (Hoshino et al. 2018b) (Fig. 3b), and dracolactams A–B (**21**, **22**) in *Micromonospora wenchangensis* HEK-797 (Hoshino et al. 2017) (Fig. 3c). Notably, niizalactams, mirilactams, and dracolactams did not exhibit antimicrobial or cytotoxic activity. These isolated compounds contain distinct cyclization patterns, despite originating from similar common precursors (Fig. 3d). Although some reactions may occur spontaneously, this suggests that enzymes involved in *E*/*Z* double bond isomerization, epoxidation and its induced cyclization, as well as subsequent Diels–Alder cycloaddition may be activated in the cells through combined-culture stimulation (Fig. 3d). Additionally, umezawamides A and B (**23**, **24**) were discovered in *Umezawaea* sp. RD066910 co-cultured with *T. pulmonis*, with umezawamide A displaying antifungal activity, while both umezawamides A and B exhibit cytotoxicity (Hoshino et al. 2018c) (Fig. 4a). Furthermore, *Rhodococcus wratislaviensis* MACB induced the production of glycosylated polyene macrolactams (ciromicins A and B) (**25**, **26**) in *Nocardiopsis* sp. FU40 ΔApoS (Derewacz et al. 2015) (Fig. 4b), a strain that cannot biosynthesize glycosylated type-I polyketide apoptolidin. This disruption may prevent competition for biosynthetic precursors, such as malonyl-CoA.

#### Siderophores

Iron is one of the most abundant elements on Earth. It primarily exists as Fe(OH)_3_, which has low water solubility. To adapt to iron-starvation conditions, microbes produce iron-chelating metabolites known as siderophores (Schalk 2025). Siderophores play a crucial role in cellular iron storage and are secreted to facilitate iron uptake from the environment. Iron competition via siderophore production is well-documented in bacterial interactions (Traxler et al. 2013; Zang et al. 2025).

Several siderophores have been isolated from combined-cultures. *Catenuloplanes* sp. RD067331 produces catenulobactins A and B (**27**, **28**) (Hoshino et al. 2018a) (Fig. 4c). Catenulobactin B, which contains *N*-hydroxyornithine and oxazoline moieties, exhibits Fe(III)-chelating activity and moderate cytotoxicity. *Streptomyces davawensis* JCM 4913 produces desferrioxamines I_2a_, I_1a_, and I_1b_ (**29–31**) (Hagihara et al. 2018) (Fig. 4d). In these compounds, the N-terminal of desferrioxamine B is substituted with an unusual heterocyclic unit—either 2-methyl-4-propyloyl-pyrrole (I_1b_) or 2-methyl-2-hydroxy-3-oxo-4-propyloyl-2-pyrroline (I_1a_). These heterocyclic ring-fused desferrioxamines exhibit Fe(III)-chelating activity. In *S. davawensis*, inactivation of a *creD* homolog gene—known to be involved in nitric oxide generation from aspartate (Sugai et al. 2016)—abolished heterocyclic ring derivatization, although the exact mechanism in the biosynthesis of the heterocyclic unit remains unknown.

#### Siderophore antibiotic hybrid

*Streptomyces hygroscopicus* HOK021 produces harundomycin A (**32**) in a combined-culture (Asamizu et al. 2022) (Fig. 4e). Harundomycin A is a conjugate of the 2,4-dihydroxy-3-aminobenzoate moiety of platensimycin and *N*,*N*′-bis(2,3-dihydroxybenzoyl)-*O*-seryl-cysteine (bisDHBA-Ser-Cys) via a thioester linkage. Platensimycin is a unique inhibitor of bacterial FabF (ketosynthase) in fatty acid biosynthesis. However, modification with the bulky bisDHBA-Ser-Cys moiety results in reduced bioactivity against methicillin-resistant *Staphylococcus aureus* (MRSA) and vancomycin-resistant *Enterococcus* (VRE). The conjugation of antibiotic and siderophore (known as a sideromycin) functions as a"Trojan Horse"strategy where the antibiotic mimics a siderophore to exploit bacterial siderophore transporters for active import (Miao et al. 2025). Although the biosynthetic mechanism and biological function of harundomycin A remain unclear, this modification may also serve as a detoxification strategy for platensimycin in the MACB partner bacterium, as well as support the active import of the antibiotic warhead.

#### Cyclic peptides and others

**Fig. 5:**
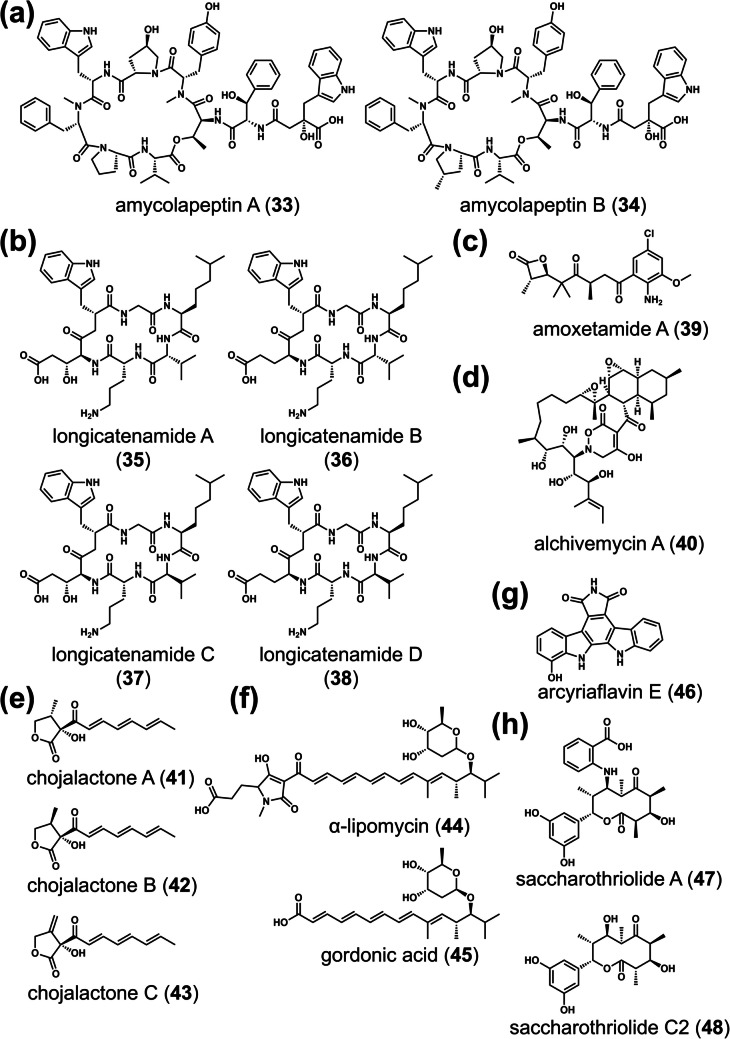
Chemical structures of diverse SMs induced in actinobacteria during co-culture with MACB. Chemical structures of amycolapeptins A–B (**a**), longicatenamides A-D (**b**), amoxetamide A (**c**), alchivemycin A (**d**), and chojalactone A–C (**e**) from combined-culture with *T. pulmonis*. Chemical structures of α-lipomycin and gordonic acid from co-culture of *Streptomyces tendae* KMC006 and *Gordonia* sp. KMC005 (**f**). Chemical structures of arcyriaflavin E (**g**) and saccharothriolide A and C2 (**h**) from combined-culture with *T. pulmonis*

*Streptomyces* sp. KUSC_F05 produces head-to-tail cyclic peptides, longicatenamides A–D (**35–38**), which are likely synthesized by nonribosomal peptide synthetases (NRPS) (Jiang et al. 2021) (Fig. 5b). Among these, longicatenamide A exhibited weak antibacterial activity against *Bacillus subtilis*. Although *Amycolatopsis* sp. 26–4 produces cyclic depsipeptides (amycolapeptins A–B) (**33**, **34**) that are likely synthesized by NRPS (Pan et al. 2021) (Fig. 5a), their bioactivity is unreported. Additionally, *Amycolatopsis* sp. 26–4 produces amoxetamide A (**39**), a β-lactone-containing compound, in combined-culture (Pan et al. 2024; Tokuda et al. 2025) (Fig. 5c). Amoxetamide A induces anoikis, a form of programmed cell death triggered by cell detachment from the extracellular matrix.

Aside from the metabolites mentioned above, natural products with diverse chemical structures have been isolated. *Streptomyces endus* S522 (= *Streptomyces* sp. TP-A0867) produce the nonribosomal peptide (NRP)-polyketide (PK) hybrid alchivemycin A (**40**) in combined-culture (Igarashi et al. 2010; Onaka et al. 2011; Komaki et al. 2016) (Fig. 5d). Alchivemycin A exhibits potent antibacterial activity against *Micrococcus luteus*, with a minimum inhibitory concentration (MIC) of 50 nM. *Streptomyces* sp. CJ-5 produces butanolide derivatives (chojalactones A–C) (**41–43**) which demonstrate moderate cytotoxicity (Hoshino et al. 2015b) (Fig. 5e). Gordonic acid (**45**) is produced in the co-culture of *Gordonia* sp. KMC005 and *Streptomyces tendae* KMC006 (Park et al. 2017) (Fig. 5f). Gordonic acid lacks the tetramic acid (2,4-pyrrolidinedione) ring and appears to be an intermediate of an antibiotic α-lipomycin (**44**) produced by *S. tendae* KMC006, with its tailoring step likely inhibited by co-culture stimuli. *Streptomyces cinnamoneus* NBRC 13823 produces indolocarbazole arcyriaflavin E (**46**), which is cytotoxic (Hoshino et al. 2015c) (Fig. 5g). *Saccharothrix* sp. A1506 produces saccharothriolide analog C2 (**48**) along with saccharothriolide A (**47**) as its major compound, which is cytotoxic (Jiang et al. 2019) (Fig. 5h).

### Uniting combined-culture with other genetic methods

Biosynthetic genes for SMs are typically clustered together in the genome, forming SM-BGCs (Gavriilidou et al. 2022). Heterologous expression of SM-BGCs has broad applications, including biosynthetic pathway analysis, combinatorial biosynthesis through gene addition or deletion, and metabolic engineering for high-yield production (Rutledge and Challis 2015). Evaluating strategies to combine co-culture experiments with genetic methods such as heterologous expression is important for optimizing SM production. Enhanced SM production was observed when *S. lividans* TK23 (harboring a heterologous BGC) was combined-cultured with *T. pulmonis* (Onaka et al. 2015) (Table 2). For example, heterologous expression of the linear azole-containing RiPP goadsporin (originally from *Streptomyces* sp. TP-A0584), the tryptophan derived indolocarbazole staurosporine (originally from *Streptomyces* sp. TP-A0274), and another tryptophan derived indolocarbazole rebeccamycin (originally from *Lechevalieria aerocolonigenes* ATCC 39243) in *S. lividans* TK23 overproduced these metabolites (Onaka et al. 2015). Moreover, this approach led to the discovery of goadsporin B and C, which are key biosynthetic intermediates that helped elucidate the biosynthetic pathway, particularly the role of glutamination in the dehydralanine formation of RiPP biosynthesis (Ozaki et al. 2016). Additionally, heterologous expression of the SM-BGCs for streptoaminals and 5aTHQs resulted in a phenomenon similar to that observed in the original producing strain HEK616, where co-cultivation induced the production of 5aTHQs as described earlier (Ozaki et al. 2019).

**Table 2:** Enhanced natural products in heterologous expression combined-culture

induced natural products	BGC	heterologous expression host	inducer strain	references
goadsporin A-C	*god* BGC from *Streptomyces* sp. TP-A0584	*S. lividans* TK23	Tp	(Onaka et al. 2015; Ozaki et al. 2016)
staurosporin	*sta* BGC from *Streptomyces* sp. TP-A0274	*S. lividans* TK23	Tp	(Onaka et al. 2015)
rebeccamycin	*reb* BGC from *Lechevalieria aerocolonigenes* ATCC 39243	*S. lividans* TK23	Tp	(Onaka et al. 2015)
streptoaminals (**12**–**14**)	*stm* BGC from *Streptomyces nigrescens* HEK616	*S. lividans* TK23	Tp	(Ozaki et al. 2019)
5-alkyl-1,2,3,4-tetrahydroquinolines (**4**–**11**)	*stm* BGC from *Streptomyces nigrescens* HEK616	*S. lividans* TK23	Tp	(Ozaki et al. 2019)

Tp: *Tsulamurella pulmonis* TP-B0596

### Ecological relevance of actinobacteria vs. MACB interaction

To investigate the significance of interactions between SM-producing actinomycetes and MACB, the characteristics of bacteria isolated from environmental samples (including soil) were evaluated (Kato et al. 2022). Kato et al. (2022) isolated actinomycetes from soil samples collected on Hegura Island, Ishikawa, Japan, an island known as a stopover point for migratory birds. Some stock samples contained mixtures of two or more bacterial species, which are generally considered contamination. However, careful evaluation discovered that several samples contained actinomycetes and MACB (Kato et al. 2022). When the natural mixture was co-cultured in liquid media, coaggregation was observed between the actinomycetes and MACB as well as the induced production of SMs, suggesting a potential ecological significance in interaction between actinomycetes and MACB involving secondary metabolism (Asamizu et al. 2015; Kato et al. 2022).

## Conclusion remark

Recent studies demonstrate that microbial interactions can serve as physiological triggers to activate silent BGCs and lead to the production of novel natural products through bacterial interplay. Therefore, leveraging the communication between actinomycetes and other organisms presents a promising strategy for addressing these challenges. However, much of the genetic basis underlying these interactions remains unexplored. Deciphering these molecular mechanisms could be the key to overcoming these hurdles, potentially unlocking new avenues for natural product drug discovery.

## Data Availability

No datasets were generated or analysed during the current study.
